# Hematocrit-adjusted tacrolimus levels are associated with acute kidney injury but not rejection early after liver transplantation

**DOI:** 10.3389/frtra.2026.1878595

**Published:** 2026-07-03

**Authors:** Trana Hussaini, Daljeet Chahal, Vladimir Marquez, Tianyi Wen, Monica Dahiya, Eric M. Yoshida, Michael R. Law

**Affiliations:** 1 Pharmaceutical Sciences, University of British Columbia, Vancouver, BC, Canada; 2 Division of Gastroenterology, Faculty of Medicine, University of British Columbia, Vancouver, BC, Canada; 3 Gastroenterology and Hepatology Research Program, Vancouver, BC, Canada; 4 Department of Community Health Sciences, Cumming School of Medicine, University of Calgary, Calgary, AB, Canada; 5 Centre for Health Policy, O’Brien Institute for Public Health, University of Calgary, Calgary, AB, Canada

**Keywords:** acute kidney injury, drug monitoring, hematocrit, immunosuppression, liver transplantation, rejection, tacrolimus

## Abstract

**Introduction:**

Tacrolimus (Tac) is highly bound to erythrocytes, with less than 1% present in the pharmacologically active unbound fraction. In anemia, whole-blood trough concentrations may underestimate effective exposure. Hematocrit (Hct) adjustment has been proposed but clinical outcome data are limited.

**Methods:**

We conducted a single-center retrospective cohort study of adult liver transplant recipients (2018 to 2022) to examine the relationship between Hct-adjusted Tac and early clinical outcomes. Tac troughs, Hct, and serum creatinine were collected for 90 days. Hct-adjusted Tac was calculated as (0.45 ÷ Hct) × total Tac. The difference between adjusted and measured Tac (delta Tac) was used to represent anemia-related underestimation and modeled as a time-varying covariate in Cox regression for acute kidney injury (AKI) and biopsy-proven T cell-mediated rejection (TCMR), adjusting for established risk factors.

**Results:**

Among 344 recipients (median age 59 years; 62% male), TCMR occurred in 15.7% and AKI in 69.8%. Delta Tac was not associated with TCMR (HR 0.93, 95% CI 0.77 to 1.14) but was associated with higher AKI hazard (HR 1.15 per ng/mL, 95% CI 1.08 to 1.24).

**Discussion:**

These findings suggest that divergence between Hct-adjusted and measured Tac is associated with increased AKI risk without a corresponding signal for rejection.

## Introduction

Acute kidney injury (AKI) has emerged as a common and critical complication after liver transplantation, affecting up to 60% of recipients (1–3). Even mild or transient AKI episodes early post transplantation have been shown to significantly worsen long-term outcomes, including chronic kidney disease (CKD), graft loss, and mortality (3, 4). Indeed, liver transplant recipients exhibit the highest incidence of CKD among non-renal solid organ transplants and progression to advanced CKD increases mortality fourfold (5). Although multiple perioperative factors contribute to AKI, exposure to calcineurin inhibitors remains a major cause (2). Kidney-sparing protocols including tacrolimus minimization and delayed initiation strategies, first reported in the early 2000s have achieved modest renal preservation at one year, yet AKI rates remain unacceptably high, underscoring the need for additional optimization strategies (6, 7).

One potential but underrecognized pharmacokinetic determinant of tacrolimus exposure is hematocrit. Tacrolimus distributes extensively into red blood cells, with a blood-to-plasma ratio of approximately 50 at a hematocrit of 45% and less than 1% of the drug existing in unbound form (8). Consequently, low hematocrit leads to lower whole-blood tacrolimus concentrations and may be misinterpreted as increased clearance (9). In anemic patients, the proportion of unbound (pharmacologically active) tacrolimus is markedly elevated compared with patients with normal hematocrit (10). Thus, in the setting of anemia which is seen in more than 50% of liver transplant recipients postoperatively, whole-blood tacrolimus levels may underestimate unbound exposure, potentially predisposing to tacrolimus related nephrotoxicity despite apparently therapeutic levels (11, 12).

While measurement of unbound or plasma tacrolimus concentrations would most accurately reflect pharmacologically active exposure, such assays are technically demanding, costly and not currently available for routine clinical use. As a practical alternative, hematocrit-adjusted tacrolimus concentrations have been proposed to approximate unbound exposure (9, 13). For instance, a hematocrit-corrected tacrolimus concentration formula (C_standardized_ = C_total_ × 0.45/Hct) has been suggested in kidney transplant recipients. Yet, the formulas have not been systematically evaluated for their correlation with clinical outcomes such as rejection or kidney injury.

To examine whether anemia-related underestimation of tacrolimus exposure is clinically meaningful rather than purely pharmacokinetic, we evaluated the association between the degree of underestimation, operationalized as delta Tac (the difference between Hct-adjusted and measured whole-blood tacrolimus) and clinical outcomes. Because larger delta Tac values reflect progressively greater underestimation of tacrolimus exposure by conventional whole-blood monitoring, we hypothesized that greater divergence between hematocrit-adjusted and measured tacrolimus concentrations would be associated with a higher risk of acute kidney injury. In contrast, we did not anticipate a corresponding increase in allograft rejection because Hct-adjusted tacrolimus concentrations in anemic patients may remain within a range sufficient for immunosuppressive efficacy despite apparently low measured whole-blood concentrations. Direct evaluation of this hypothesis would require a prospective study comparing dosing guided by hematocrit-adjusted concentrations with standard whole-blood Tac monitoring. However, in the absence of clinical outcome data supporting the safety of hematocrit-guided dosing, an observational approach represents an appropriate initial step in evaluating the clinical relevance of hematocrit-adjusted tacrolimus exposure.

In this retrospective cohort study, we examined the relationship between anemia-related underestimation of tacrolimus exposure and clinical outcomes in liver transplant recipients. We evaluated delta Tac, defined as the difference between hematocrit-adjusted and measured whole-blood tacrolimus concentrations, as the primary exposure metric and assessed its association with acute kidney injury and biopsy-proven rejection.

## Methods

### Study design and population

We conducted a single center, retrospective cohort study including all adult patients (≥18 years) who underwent liver transplantation between January 1, 2018, and December 31, 2022 at Vancouver General Hospital. We excluded patients who died within the first postoperative week and those who required renal replacement therapy within two weeks before or after transplantation. Recipients of combined liver-kidney transplants were also excluded. Eligible patients were required to have received and maintained tacrolimus-based immunosuppression for at least the first 3 months post-transplant.

Our center's early immunosuppression protocol consists of triple therapy with corticosteroids, mycophenolate mofetil, and tacrolimus. Patients at increased risk for peri-operative AKI are managed with basiliximab induction and delayed tacrolimus initiation (typically to postoperative day 5). Corticosteroids are administered as IV methylprednisolone for 5 days, followed by an oral prednisone taper over 4 months. Mycophenolate mofetil 1,000 mg twice daily is initiated post-transplant. Tacrolimus is initiated per protocol at 0.03–0.05 mg/kg twice daily and titrated to target whole-blood trough concentrations. During the first month post-transplant, target trough levels are 8–10 ng/mL in patients with preserved renal function and 6–8 ng/mL in those with kidney dysfunction. In months 2 and 3, targets are reduced to 7–9 ng/mL and 5–7 ng/mL, respectively, with lower targets generally applied in patients with impaired renal function.

All patients were followed longitudinally after transplant, with outcomes assessed at prespecified time points within the first year. This research was conducted in accordance with the Declarations of Helsinki and Istanbul. The study protocol was approved by the University of British Columbia Clinical Research Ethics Board (H22-02983). Given the retrospective nature of the study and the use of anonymized data, the requirement for informed consent was waived by the Clinical Research Ethics Board.

### Clinical data

Data for this study were obtained in part from the British Columbia Transplant (BCT) Registry, a provincial database that prospectively captures demographic, clinical and transplant-related information on all solid organ recipients in British Columbia. Data were accessed for secondary use under institutional ethics approval.

Patient demographics, preoperative characteristics, intraoperative parameters, graft features and postoperative laboratory values were retrieved from the BCT Registry. Additional postoperative variables, including immunosuppressive regimens and clinical endpoints, were collected from medical records.

A dedicated research database was developed using REDCap (REDCap Consortium, Vanderbilt University, Nashville, TN), a secure, web-based application designed to support data capture for research studies.

All tacrolimus trough concentrations obtained within the first 90 days post-transplant were collected along with corresponding daily tacrolimus doses, serum creatinine and hematocrit values. Hematocrit-adjusted tacrolimus concentrations were calculated using the following equation: Hct-adjusted Tac = (0.45 ÷ Hct) × Total Tac.

The difference between the Hct-adjusted and total tacrolimus levels (Delta Tac = Hct-adjusted Tac – Total Tac) represents the difference between the laboratory-reported whole-blood tacrolimus level and the value standardized to a hematocrit of 0.45 (i.e., the underestimation attributable to anemia).

### Clinical endpoints

Laboratory results and pathology reports were reviewed for evidence of AKI and TCMR. AKI, acute kidney disease (AKD) and chronic kidney disease (CKD) were defined according to KDIGO criteria (14, 15). Baseline renal function was defined as the lowest serum creatinine within 3 months pre-transplant. Immune-mediated liver disease included any patient with history of autoimmune liver disease, primary biliary cholangitis or primary sclerosing cholangitis.

At our center, liver biopsies are performed only for clinical indication. All biopsy-proven TCMR episodes were graded according to the Banff classification (16) and only those with a rejection activity score ≥3 were included. Early acute TCMR was defined as an episode occurring within 90 days post-transplant.

Early allograft dysfunction was defined as one of the following: bilirubin ≥ 171 μmol/L on postoperative day 7, an international normalized ratio (INR) ≥ 1.6 on postoperative day 7, or alanine or aspartate aminotransferase (ALT/AST) > 2,000 IU/L within the first 7 days after surgery (17).

### Statistical analysis

Continuous variables are summarized as mean ± SD if approximately normal or median (IQR) if skewed; categorical variables as counts and percentages. Bivariable associations with the primary outcomes (AKI and TCMR) and patient characteristics were assessed using the Mann Whitney U test for continuous variables and Chi-Square test or Fisher's exact tests for categorical variables as appropriate. All tests were two-sided with *P* < 0.05 considered statistically significant.

Covariates were selected *a priori* based on established clinical relevance and published literature. In addition, variables demonstrating statistically significant associations with the outcomes in bivariate analyses were considered for inclusion to account for potential confounding.

Multivariable logistic regression was used to identify independent risk factors for TCMR and AKI, with results reported as odds ratios and 95% confidence intervals. *A priori* covariates included in the TCMR model were recipient age >60 years, etiology of end stage liver disease (immune-mediated versus nonimmune-mediated), CMV donor recipient mismatch status, induction immunosuppression, pre-transplant chronic kidney disease and total estimated intra-operative blood loss.

For AKI, multivariable logistic regression included the following *a priori* covariates: recipient age >60 years, sex, etiology of liver disease, history of AKI, pre-transplant chronic kidney disease, serum creatinine at transplant, intra-operative transfusions and blood loss, induction immunosuppression and early allograft dysfunction.

To evaluate the impact of hematocrit-adjusted tacrolimus exposure on clinical outcomes, time-to-event analyses were performed for AKI and TCMR. Cox proportional hazards models were fitted with Delta Tac defined as the difference between the hematocrit-adjusted tacrolimus concentration and the measured whole-blood tacrolimus concentration (Hct-adjusted Tac minus Total Tac). Delta Tac was modeled as a time-varying covariate to account for changes in exposure over time. Models were adjusted for baseline recipient characteristics and established clinical confounders of AKI and TCMR. Proportional hazards assumptions were assessed using Schoenfeld residuals, with results reported as hazard ratios and 95% confidence intervals.

Analyses were performed using R (R Foundation for Statistical Computing) with the dplyr, tidyr, purrr, survival and mice packages.

## Results

Between 2018 and 2022, 413 adults underwent liver transplantation. Sixty-nine patients were excluded: five died within one week of transplant, one had incomplete data and 63 required dialysis in the two weeks before or after transplant. The final analytic cohort included 344 patients with a median age of 59.0 years (IQR 49–64.5) and 62.2% were male. Median MELD-Na was 18 (IQR 13–24) and the median Child-Pugh score was 9 (IQR 7–10). Alcohol-related liver disease (22.7%) and MASLD (14.2%) were the most common etiologies and 23.8% of recipients had hepatocellular carcinoma. Median baseline serum creatinine was 80 µmol/L (IQR 66–96) and increased to 87.5 µmol/L (IQR 71.5–113.5) at the time of transplantation. Pre-transplant AKI occurred in 19.2% of patients, and chronic kidney disease was present in 21.2%. At one month post-transplant (±7 days), anemia was common with a median hematocrit of 0.32 (IQR 0.28–0.35). Detailed recipient, donor and operative characteristics are summarized in Table 1.

**Table 1:** Recipient and donor baseline characteristics, intra-operative and post-operative variables.

Variable Median [IQR], N (%)	*N* = 344
Recipient Characteristics	
Age, years	59.0 [49.0-64.5]
Male sex	214 (62.2%)
MELD Score	15 [10-20]
MELD-Na Score	18 [13-24]
Child-Pugh Score	9 [7-10]
Weight, kg	72.0 [62.0-85.0]
BMI	24.8 (22.0-28.3]
CMV - Positive	227 (66.0%)
Liver Disease Severity	
Ascites	
None	88 (25.6%)
Diuretic responsive	159 (46.2%)
Diuretic refractory	97 (28.2%)
Hepatic Encephalopathy	
None	149 (43.3%)
West Haven Criteria Grade 1	126 (36.6%)
West Haven Criteria Grade 2	49 (14.25%)
West Haven Criteria Grade 3	15 (4.4%)
West Haven Criteria Grade 4	5 (1.5%)
Fulminant liver failure	8 (2.3%)
PVT before transplant (Yes %)	52 (15.1%)
Primary ESLD Etiology	
ALD	78 (22.7%)
MASLD	49 (14.2%)
HCV	14 (4.1%)
HBV	5 (1.5%)
PSC	32 (9.3%)
PBC	20 (5.8%)
AIH	16 (4.7%)
HCC	82 (23.8%)
Other	48 (14.0%)
Baseline SCr, μmol/L	80 [66-96]
SCr at transplant, μmol/L	87.5 [71.5-113.5]
eGFR at transplant, mL/min	79.0 [58.0-96.5]
AKI Pre-Transplant	66 (19.2%)
KDIGO Stage 1	34 (9.9%)
KDIGO Stage 2	16 (4.7%)
KDIGO Stage 3	15 (4.4%)
CKD Pre-Transplant	73 (21.2%)
Stage 3	64 (18.6%)
Stage 4	9 (2.6%)
Stage 5	0 (0%)
Donor Characteristics	
Donor age, years (Mean ± SD)	39.0 [28.0-53.0]
Type of donation	
DCC	43 (12.5%)
DNC	301 (87.5%)
CMV seropositive	183 (53.2%)
Intra-Operative Variables	
Operative time, hr	5.9 [4.8, 6.8]
Cold ischemia time, min	276 [236 - 336]
Re-Warm Time, min	41.5 [33.5, 53]
Donor warm ischemic time, min (DCC only)	23 [19, 27]
Surgical technique	
Piggyback	215 (62.5%)
Classic	129 (37.5%)
Biliary reconstruction	
Duct to duct	280 (81.6%)
Roux en Y Hepaticojejunostomy	63 (18.4%)
Total estimated blood loss, mL	2,000 [1,244-3,225]
Intra-operative transfusions	
Fibrinogen (g)	4 [0-12]
PRBC (units)	2 [0-4]
PRBC ≥4 units, n (%)	129 (37.7%)
FFP (Units)	0 [0-1]
Platelets (Units)	1 [0-2)
Cell Saver/autologous blood, mL	570 [330-1,050]
Post Reperfusion Syndrome (PRS)	18 (5.2%)
Post-Operative variables	
CMV mismatch (D+/R-)	61 (17.7%)
Induction Immunosuppression	
** **Basiliximab	217 (63.1%)
** **ATG	1 (0.3%)
** **Methylprednisolone	126 (36.6%)
Delayed Tac initiation	184 (53.5%)
Peak post-op AST	949 [519-1,967]
Peak Post-op ALT	797 [418-1,500]
Early Allograft Dysfunction	92 (26.7%)

AIH, autoimmune hepatitis; AKI, acute kidney injury; ALD, alcohol-associated liver disease; ALT, alanine aminotransferase; AST, aspartate aminotransferase; CKD, chronic kidney disease; CMV, cytomegalovirus; DCC, donation after circulatory death; DNC, donation after neurologic death; FFP, fresh frozen plasma; HBV, hepatitis B virus; HCC, hepatocellular carcinoma; HCV, hepatitis C virus; KDIGO, Kidney Disease Improving Global Outcomes; MASLD, metabolic dysfunction associated steatotic liver disease; MELD, Model for End Stage Liver Disease; MELD-Na, Model for End Stage Liver Disease Sodium; PBC, primary biliary cholangitis; PRBC, packed red blood cells; PRS, postreperfusion syndrome; PSC, primary sclerosing cholangitis; PVT, portal vein thrombosis; SCr, serum creatinine.

**Figure 1:**
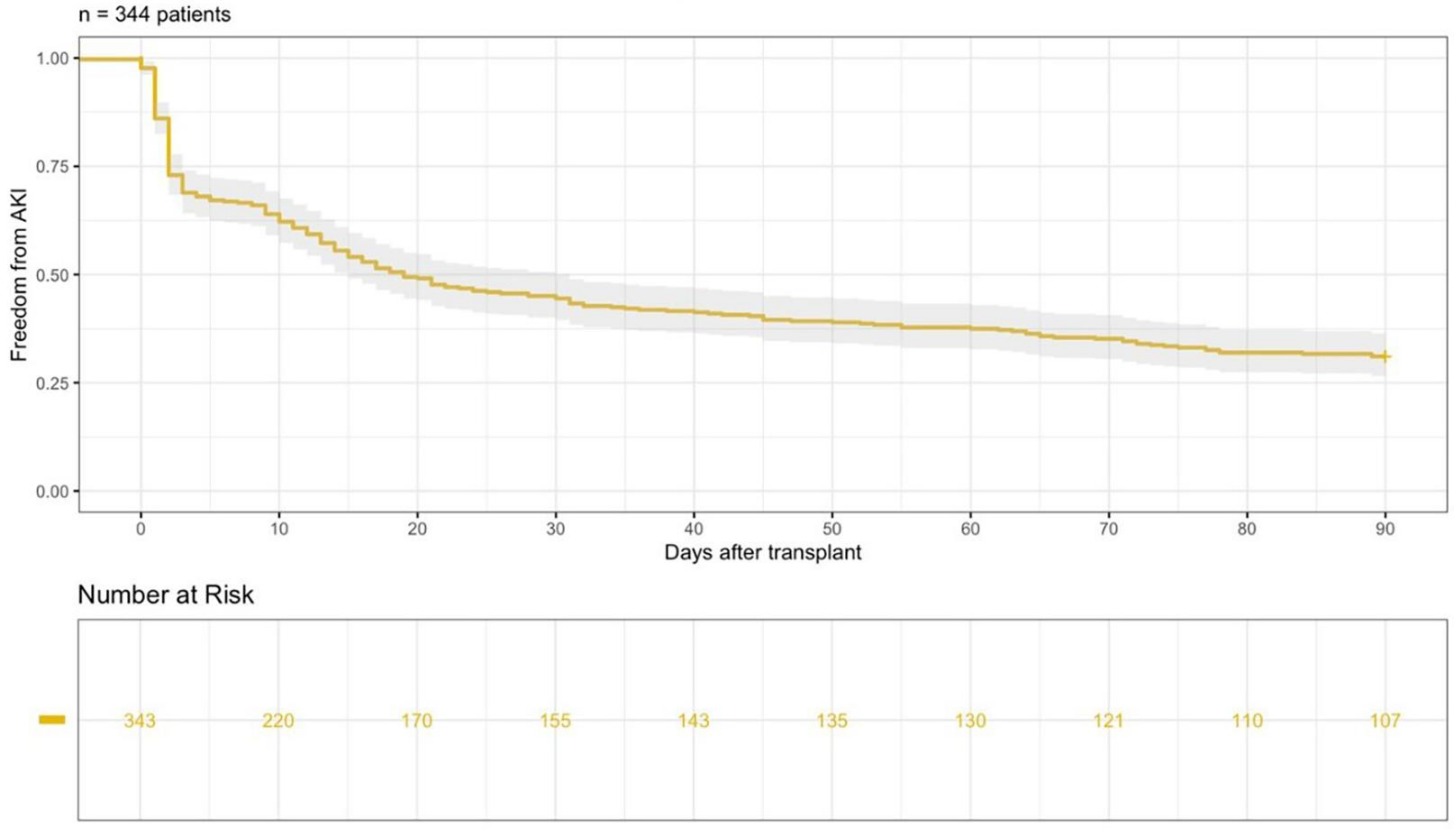
Kaplan–Meier curve of time to AKI within 90 days.

**Figure 2:**
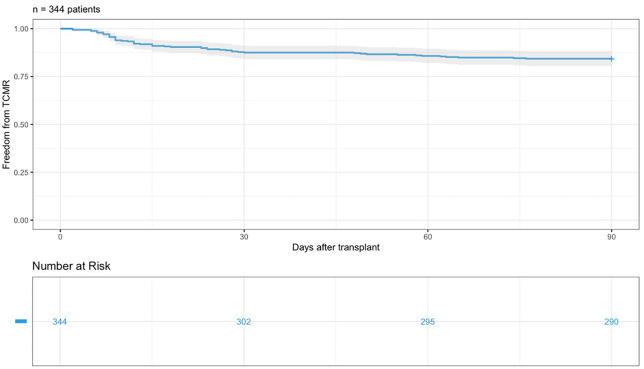
Kaplan–Meier curve of time to TCMR within 90 days.

All recipients received a standardized corticosteroid taper, tacrolimus and mycophenolate mofetil. Tacrolimus initiation was delayed in 53.5% of recipients and basiliximab induction was used in 63.1%. Early allograft dysfunction occurred in 26.7% of patients. Within 90 days post-transplant, 69.8% (240/344) of patients experienced at least one episode of AKI and nearly one third developed recurrent AKI (Figure 1; Table 2). Most AKI episodes were KDIGO stage 1; however, 159 of 240 patients with AKI progressed to acute kidney disease, indicating structural kidney damage (Table 2). By one year, more than 40% of patients (145/344) had progressed to chronic kidney disease, predominantly stage G3a or G3b (Table 2). Early TCMR occurred in 54 recipients (15.7%) within 90 days, the majority within the first month (Figure 2). Over the first year, 73 recipients (21.2%) experienced TCMR, and 3.5% had more than one episode of rejection. Most rejection episodes (53.1%) were mild with a rejection activity index of 3-4/9.

**Table 2:** Summary of AKI, AKD and CKD outcomes (*N* = 344).

Characteristic	N (%)
Patients with at least one AKI episode	240 (69.8%)
AKI Stage 1	184 (76.7%)
AKI Stage 2	46 (19.2%)
AKI Stage 3	10 (4.1%)
Patients with multiple episodes of AKI	109 (31.7%)
AKD	159 (46.2%)
Progression to CKD	145 (42.2%)
CKD G3a	93 (27.0%)
CKD G3b	39 (11.3%)
CKD G4	12 (3.5%)
CKD G5	1 (0.3%)

AKI, acute kidney injury; AKD, acute kidney disease; CKD, chronic kidney disease.

**Figure 3:**
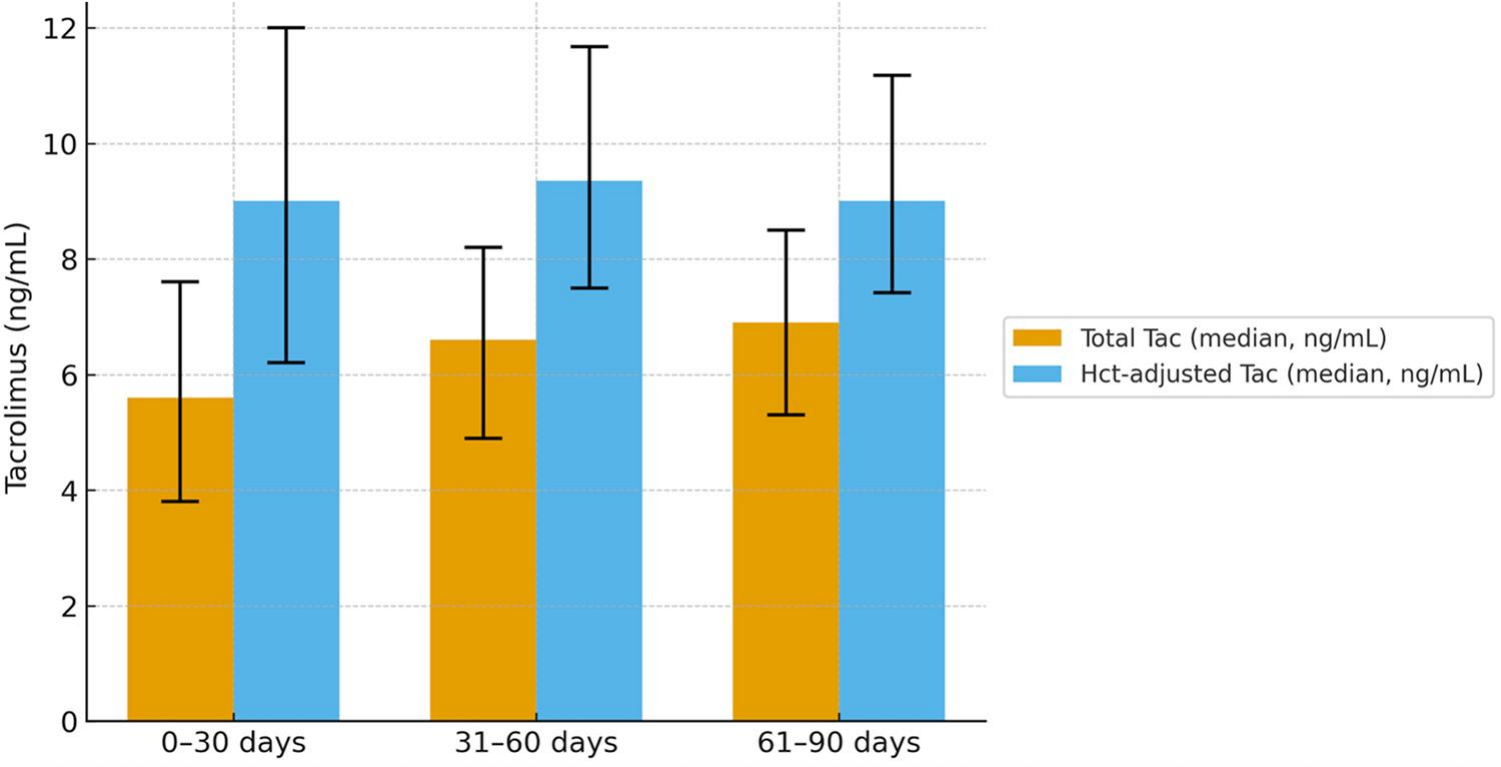
Total and Hct-adjusted tacrolimus trough levels.

Whole-blood tacrolimus trough concentrations were below conventional targets in the first month and subsequently increased to within target by months 2 to 3, reflecting the kidney-sparing strategy used in response to high incidence of AKI in this cohort. In contrast hematocrit-adjusted concentrations remained stable at approximately 9 ng/mL and within target throughout. The divergence between whole-blood and hematocrit-adjusted tacrolimus concentrations was greatest early post-transplant and diminished over time, consistent with improving hematocrit levels. (Figure 3).

In multivariable logistic regression, immune-mediated liver disease (OR 1.90; 95% CI 1.01–3.62; *P* = 0.048) and CMV D+/R– mismatch (OR 2.40; 95% CI 1.30–4.59; *P* = 0.005) were associated with higher odds of TCMR, whereas induction immunosuppression was associated with lower odds (OR 0.38; 95% CI 0.22–0.67; *P* = 0.0008) (full model parameters shown in Supplementary Table S1). For AKI, higher serum creatinine at transplantation was associated with increased odds (OR 1.01 per µmol/L increase; 95% CI 1.002–1.020; *P* = 0.014), MELD-Na demonstrated an inverse association (OR 0.94; 95% CI 0.898–0.985; *P* = 0.009) and induction immunosuppression remained protective (OR 0.34; 95% CI 0.19–0.62; *P* = 0.0004) (full model parameters shown in Supplementary Table S2).

Time-to-event analyses using time-varying Cox proportional hazards models evaluated the association between tacrolimus exposure parameterized as delta Tac and the incidence of AKI and TCMR. Delta Tac was not associated with time to TCMR (HR 0.93; 95% CI 0.77–1.14; *P* = 0.50). In contrast, each 1 ng/mL increase in delta Tac was associated with a 15% higher hazard of AKI (HR 1.15; 95% CI 1.08–1.24; *P* < 0.001) after adjustment for established risk factors (Tables 3, 4).

**Table 3:** Multivariate Cox hazard regression model for the association between TCMR and potential confounders, *N* = 344 .

Variable	Hazard Ratio	95% CI	*p*-value
Delta Tac	0.93	(0.77–1.14)	0.499
Recipient Age >60	0.74	(0.36–1.50)	0.400
Sex (female)	1.00	(0.49–2.05)	0.997
Immune-mediated Liver Disease	0.78	(0.34–1.81)	0.566
Induction Immunosuppression (Yes)	0.70	(0.34–1.45)	0.339
CMV mismatch (D+/R-)	1.21	(0.57–2.59)	0.617
Early Allograft Dysfunction	0.54	(0.21–1.43)	0.217
Surgical Blood Loss (mL)	1.0	(1.000, 1.000)	0.921

**Table 4:** Multivariate Cox hazard regression model for the association between AKI and potential confounders, *N* = 344.

Variable	Hazard Ratio	95% CI	*p*-value
**Delta Tac**	**1**.**15**	(1.08–1.24)	**<0**.**001**
Recipient Age >60	0.87	(0.61–1.25)	0.447
PRBC ≥4 units	1.06	(0.69–1.63)	0.779
Sex (female)	0.86	(0.58–1.29)	0.471
MELD-Na Score	0.98	(0.96–1.01)	0.249
Serum Creatinine at Transplant, μmol/L	1.003	(1.00, 1.01)	0.697
Cold ischemia times (min)	1.000	(1, 1.001)	0.437
Early Allograft Dysfunction	0.71	(0.44–1.14)	0.15
Fulminant liver failure	1.44	(0.31–6.63)	0.639
Induction Immunosuppression	0.98	(0.63–1.53)	0.936

## Discussion

Our findings indicate that greater divergence between hematocrit-adjusted and measured tacrolimus concentrations, consistent with anemia-related underestimation of tacrolimus exposure, is associated with an increased risk of acute kidney injury without a corresponding increase in rejection. These observations highlight the potential clinical relevance of hematocrit-adjusted tacrolimus monitoring and suggest that patients with whole-blood therapeutic troughs may nonetheless experience higher effective exposure.

The differential association of delta Tac with acute kidney injury but not with T cell-mediated rejection provides important insight into the clinical relevance of hematocrit-adjusted tacrolimus exposure. By definition, higher delta Tac reflects greater divergence between measured whole-blood and hematocrit-adjusted concentrations, consistent with underestimation of pharmacologically relevant exposure in the setting of anemia. The observed association between higher delta Tac and increased risk of acute kidney injury is therefore consistent with a model in which patients with larger divergence experience relatively higher effective tacrolimus exposure despite therapeutic or low whole-blood trough concentrations.

In contrast, no association was observed between delta Tac and rejection. If whole-blood tacrolimus concentrations were the primary determinant of immunologic protection, lower measured trough levels, and thus higher delta Tac, would be expected to be associated with an increased risk of rejection. However, this relationship was not observed despite whole-blood tacrolimus trough concentrations frequently remaining below conventional institutional targets during the early post-transplant period because of kidney-sparing immunosuppression strategies used in response to the high incidence of AKI in this cohort. Tacrolimus demonstrates a nonlinear exposure-response relationship for calcineurin inhibition, with an estimated IC₅₀ of approximately 9 ng/mL (18). Below this threshold, incremental increases in tacrolimus exposure are expected to produce clinically meaningful increases in calcineurin inhibition, whereas additional increases beyond this range may yield diminishing incremental immunosuppressive benefit. In our cohort, median whole-blood tacrolimus trough concentrations during the early post-transplant period were approximately 6 to 7 ng/mL, while hematocrit-adjusted tacrolimus concentrations remained relatively stable at approximately 9 ng/mL. Under the assumption that whole-blood tacrolimus concentrations accurately reflect biologically active exposure, and given that whole-blood tacrolimus concentrations in our cohort were generally below the estimated IC_50_, patients with larger Delta Tac values would be expected to have progressively lower effective tacrolimus exposure and therefore a higher risk of rejection. However, this relationship was not observed. On the other hand, hematocrit-adjusted tacrolimus concentrations in our cohort approximated 9 ng/mL, near the estimated plateau region of the tacrolimus exposure-response relationship for calcineurin inhibition. Under this framework, additional increases in tacrolimus exposure beyond this range may not be expected to produce substantial additional immunosuppressive benefit, which could explain the absence of a measurable association between Delta Tac and rejection risk.

Taken together, these findings raise the possibility that hematocrit-adjusted tacrolimus concentrations may better approximate pharmacologically active tacrolimus exposure during the anemic early post-transplant period. This may explain why immunologic control appeared preserved despite lower measured whole-blood concentrations.

These data offer a potential explanation for the persistently high burden of early acute kidney injury after liver transplantation even in the era of kidney-sparing immunosuppression. In the early post-operative period, anemia is common and reliance on whole-blood tacrolimus troughs may systematically underestimate exposure.

Although prior pharmacokinetic studies demonstrated the impact of anemia on whole blood tacrolimus levels and proposed correction formulas (13, 19), patient-level outcome data linking hematocrit-corrected exposure to clinical endpoints have been limited. In this study, time-varying analyses showed an association between delta tacrolimus and acute kidney injury, without a corresponding signal for increased rejection, suggesting that hematocrit-adjusted monitoring may capture clinically relevant safety information beyond a purely theoretical pharmacokinetic consideration.

In our study, induction immunosuppression was independently associated with lower risks of both AKI and TCMR, consistent with prior randomized trials (6, 7) and real-world cohorts (20–22), supporting the internal validity of our findings. The inverse association of MELD-Na with AKI likely reflects practice patterns, including greater use of induction and delayed tacrolimus initiation in higher acuity patients and residual confounding by indication. Baseline kidney function remained a robust predictor of post-transplant AKI, underscoring the multifactorial nature of renal risk in liver transplant recipients. Importantly, although the majority of AKI episodes in our cohort were mild, approximately 66% of patients subsequently developed acute kidney disease (AKD), suggesting persistence of kidney dysfunction beyond the acute phase, and approximately 40% progressed to chronic kidney disease at 1 year post-transplant. These downstream kidney outcomes suggest that a substantial proportion of AKI episodes likely reflected clinically meaningful kidney injury rather than isolated transient hemodynamic physiology alone.

The associations of immune-mediated liver disease and CMV D+/R– mismatch with higher TCMR are consistent with established immunologic risk profiles and support tailored immunosuppression and closer monitoring in these groups.

Strengths of this study include a large, contemporary liver transplant cohort managed with modern immunosuppression and current kidney-sparing practices. We applied updated KDIGO definitions for AKI, AKD, and CKD, with adjudicated clinical data to ensure accurate endpoint classification. Granular exposure assessment was achieved through comprehensive capture of tacrolimus dosing and trough concentrations linked to contemporaneous hematocrit and serum creatinine during the first 90 days post-transplant. Time-varying exposure modeling further strengthens the internal validity of this study.

Several limitations should be acknowledged. First, the retrospective design introduces the potential for residual confounding despite statistical adjustment. In particular, clinical acuity, including hospitalization, hemodynamic instability and exposure to nephrotoxic medications was not fully captured and may influence both hematocrit and the risk of acute kidney injury. Similarly, infection, systemic inflammation, hepatic dysfunction and other manifestations of acute illness may influence both tacrolimus pharmacokinetics and the risk of acute kidney injury, introducing the potential for additional residual confounding. Anemia itself is a recognized risk factor for AKI and, in the absence of adjudicated etiologies for individual AKI episodes, it remains possible that patients with greater illness severity and more pronounced anemia were inherently at higher risk of AKI, rather than anemia serving primarily as a marker of underrecognized tacrolimus exposure. In addition, because delta Tac is mathematically derived in part from hematocrit, the observed association with AKI may partially reflect postoperative anemia and overall illness severity rather than tacrolimus exposure alone. Hematocrit was not included separately in the Cox model because of collinearity with delta Tac, limiting the ability to fully disentangle these relationships. However, measured markers associated with perioperative blood loss and illness severity, including MELD-Na score, operative time, total blood loss and intra-operative transfusion requirements, were not significantly associated with AKI in the risk factor analysis. Second, tacrolimus dosing in this cohort was guided by whole-blood trough concentrations and was not modified based on hematocrit-adjusted values. Accordingly, this study does not evaluate a hematocrit-adjusted dosing strategy, does not compare alternative monitoring approaches and cannot establish a causal relationship between hematocrit-adjusted tacrolimus exposure and clinical outcomes. Third, delta Tac is derived from hematocrit standardization rather than direct measurement of unbound tacrolimus concentrations. Importantly, other determinants of tacrolimus binding, including albumin were not fully accounted for and may contribute to exposure misclassification. Fourth, the use of a point-in-time exposure metric may not fully capture cumulative or sustained tacrolimus exposure. Longitudinal measures, such as time above therapeutic thresholds based on hematocrit-adjusted concentrations may provide a more clinically relevant assessment. Finally, treatment modifications over time, including dose adjustments or temporary holds, may also influence observed exposure-outcome relationships despite the use of time-varying models.

These findings warrant evaluation in independent cohorts and replication studies are currently underway. Further work is needed to determine whether alternative exposure metrics, including hematocrit-adjusted or unbound tacrolimus concentrations can more accurately characterize risk. Prospective studies comparing hematocrit-adjusted and conventional monitoring strategies will be required to determine whether modification of tacrolimus dosing can reduce nephrotoxicity without compromising immunologic outcomes.

In summary, in a contemporary liver transplant cohort, divergence between hematocrit-adjusted and measured tacrolimus concentrations identifies patients at higher risk of acute kidney injury without a corresponding increase in rejection. These findings support the hypothesis that standard monitoring may incompletely reflect biologically relevant exposure in patients with anemia and provide a rationale for further study of hematocrit-informed approaches to tacrolimus exposure assessment.

## Data Availability

The data analyzed in this study is subject to the following licenses/restrictions: Under UBC CREB criteria, we cannot publically share patient data. Requests to access these datasets should be directed to trana.hussaini@vch.ca.
