# Supplementary material for: Hematocrit-adjusted tacrolimus levels are associated with acute kidney injury but not rejection early after liver transplantation
Source: Front Transplant. 2026 Jul 3;5:1878595. doi: 10.3389/frtra.2026.1878595 (PMC13376118; doi:10.3389/frtra.2026.1878595)
Supplement: Supplementary file 1 [file Table1.docx]

| **Table S1:** Multivariate Logistic Regression for the association between TCMR and Potential Confounders, N = 344. | | | |
| --- | --- | --- | --- |
| **Variable** | **Odds Ratio** | **95% CI** | **p-value** |
| Recipient Age >60 | 1.323 | 0.760-2.303 | 0.3230 |
| **ESLD Etiology (immune mediated)** | **1.907** | **1.005-3.616** | **0.0481** |
| **CMV mismatch** **(D+/R-)** | **2.443** | **1.302-4.586** | **0.0054** |
| **Induction Immunosuppression (Yes)** | **0.382** | **0.218-0.670** | **0.0008** |
| CKD Pre-Transplant (Yes) | 1.12 | 0.578-2.170 | 0.7378 |
| Total estimated  blood loss | 1.0 | 1.000-1.000 | 0.6299 |
| Cell Saver Blood Transfusion | 1.0 | 1.000-1.001 | 0.2828 |
| Early Allograft  Dysfunction | 2.629 | 0.312-22.178 | 0.3742 |
