# Supplementary material for: Hematocrit-adjusted tacrolimus levels are associated with acute kidney injury but not rejection early after liver transplantation
Source: Front Transplant. 2026 Jul 3;5:1878595. doi: 10.3389/frtra.2026.1878595 (PMC13376118; doi:10.3389/frtra.2026.1878595)
Supplement: Supplementary file 2 [file Table2.docx]

| **Table S2.** Multivariate Logistic Regression for the association between AKI and Potential  Confounders, N = 344. | | | |
| --- | --- | --- | --- |
| **Variable** | **Odds Ratio** | **95% CI** | **p-value** |
| Recipient Age >60 | 0.653 | 0.373-1.141 | 0.1343 |
| ESLD Etiology (Primary) | 0.869 | 0.441-1.715 | 0.6861 |
| Sex (Female) | 1.039 | 0.590-1.830 | 0.8953 |
| **MELD-Na Score** | **0.941** | **0.898-0.985** | **0.0090** |
| Ascites | 1.121 | 0.562-2.236 | 0.7451 |
| PVT (Yes) | 0.504 | 0.209-1.217 | 0.1276 |
| **SCr at transplant,**  **μmol/L** | **1.011** | **1.002-1.020** | **0.0143** |
| AKI Pre-Transplant  (Yes) | 1.017 | 0.434-2.383 | 0.9696 |
| CKD Pre-Transplant  (Yes) | 1.066 | 0.498-2.283 | 0.8684 |
| Type of donation  (NDD) | 2.361 | 0.946-5.894 | 0.0657 |
| Operative time | 0.954 | 0.762-1.195 | 0.6840 |
| Total estimated  blood loss | 1.0 | 1.000-1.000 | 0.7736 |
| PRBC ≥4 units | 0.939 | 0.819-1.078 | 0.3740 |
| Cell Saver Blood Transfusion | 1.0 | 0.999-1.000 | 0.6499 |
| CMV mismatch  (D+/R-) | 0.645 | 0.313-1.332 | 0.2361 |
| **Induction**  **Immunosuppression** **(yes)** | **0.34** | **0.188-0.617** | **0.0004** |
| Early Allograft  Dysfunction | 1.037 | 0.234-4.601 | 0.9621 |
